# Poisonous Rooms

**Published:** 1860-05

**Authors:** 


### POISONOUS ROOMS.

NOTHING short of "line upon line" is sufficient to impress great practical truths on the common mind; hence the reïteration of the fact that using wall-paper having a green color in it, especially if fuzzy, and not glazed, is immediately destructive of health, and of life itself if persisted in; as proof: H. Fulland, near Tipton, England, lately moved in a new house; all his children became curiously affected, worse at night than during the day: they were exceedingly restless; a singular twitching or jerking of the muscles, especially of the face, and general decline of health, indicated the working of some insidious agency. The physician had them promptly removed to another room, when they began at once to recover their health. On a small piece of green-colored paper on the walls of the room left, there was found on analysis enough arsenic to poison a man.

